# Serum‐Based miRNA Panel as Diagnostic Biomarkers for Hepatitis C Virus‐Induced Hepatocellular Carcinoma: A Cross‐Sectional Study

**DOI:** 10.1002/hsr2.72377

**Published:** 2026-04-15

**Authors:** Badr Alzahrani, Yasmeen Ishaq, Qaiser Farid Khan, Tawaf Ali Shah, Gamal A. Shazly, Mohammed Bourhia, Aqsa Ikram

**Affiliations:** ^1^ Department of Clinical Laboratory Sciences, College of Applied Medical Sciences Jouf University Sakaka Saudi Arabia; ^2^ Institute of Molecular Biology and Biotechnology (IMBB) University of Lahore (UOL) Lahore Pakistan; ^3^ Department of Microbiology, Ikam ul Haq Institute of Industrial Biotechnology Government College University Lahore Pakistan; ^4^ College of Agriculture Engineering and Food Science Shandong University of Technology Zibo China; ^5^ Department of Pharmaceutics, College of Pharmacy King Saud University Riyadh Saudi Arabia; ^6^ Department of Chemistry and Biochemistry, Faculty of Medicine and Pharmacy Ibn Zohr University Laayoune Morocco

## Abstract

**Background and Aims:**

Hepatocellular carcinoma (HCC) is responsible for more than 90% of primary hepatic cancer. Hepatitis C virus (HCV) infection is one of the contributing factors for HCC development. miRNAs are non‐coding RNAs and are also involved in HCV replication. Expression variability of miRNA has also been reported in various cancers, including HCC.

**Methods:**

By using different bioinformatics tools, panels of serum‐based miRNA, including *miR‐1*, *miR*−200b, *miR*−320d, miR‐346, and *miR*−451a, were selected to investigate their role in HCV and HCV‐HCC. Furthermore, RT‐PCR was employed to confirm their regulation pattern in relation to the NF‐KB and IL‐6 in various study groups, including GI=control, GII = HCV, GIII = HCV_HCC, and GIV = HCC.

**Results:**

The expression levels of miR‐1, miR‐346, and miR‐451 were significantly downregulated in GII and GIII compared with GI. However, upregulated expression was observed against miR‐200b, miR‐320d, NF‐κB, and IL‐6 in GIII when compared with GI. In GIII, miR‐451a (*r* = 0.6, *p* < 0.02) was found to have a positive association with NF‐κB, while miR‐1 (*r* = −0.7, *p* < 0.003) has a negative association with IL‐6. ROC analysis revealed that the selected miRNAs, along with their related biomarkers, have enhanced the overall sensitivity and specificity (88% and 0.6%) of the GIII group compared to the GI group.

**Conclusion:**

The selected panel of miRNAs has served as diagnostic biomarkers against HCV‐HCC.

## 1 Introduction

Hepatocellular carcinoma (HCC) is the most common type of liver cancer. It is caused by different risk factors, including alcohol consumption, hepatitis C virus (HCV), and hepatitis B virus (HBV) infection. MicroRNA (miRNA) aberrant expression has been reported in various kinds of malignancies, including HCC induced by HCV and HBV infection [1, 2, 3]. Worldwide, more than 170 million people are infected by HCV, and more than 70 million are in a chronic disease state [4]. A meta‐analysis of case‐control investigations found that patients with anti‐HCV antibodies have a 17‐fold higher risk for HCC development as compared to those individuals who did not test positive for HCV‐specific antibodies [5]. Mostly, HCV infection is asymptomatic. In 60–80% of cases, HCV infection becomes a chronic infection [4, 6]. In some people, cirrhosis remains slothful for several years; however, other individuals show the development of hepatic decompensation, HCC, and death [7].

The miRNAs are ~22‐nt long, non‐coding, endogenous RNAs that play a critical role in mRNA expression regulation by interacting with 3' UTR (untranslated region) of target mRNA [8, 9]. Several studies reported that miRNAs play a crucial role in different pathologies, including cancer, inflammatory bowel disease [10], liver disease, coronary heart disease, and metabolic disease [11]. In HCV‐HCC development, various signaling pathways, including mammalian target of rapamycin (mTOR), wingless‐type MMTV integration site family (Wnt), apoptosis, and the Mitogen‐Activated Protein Kinase (MAPK) pathway, are also disrupted by irregulated expression of miRNA [12]. HCV‐HCC infection also alters the expression of several host miRNAs, which can promote liver inflammation, fibrosis, and carcinogenesis [13]. miRNAs profoundly influence both HCV replication and the progression of HCC associated with HCV infection [14]. While several miRNAs are known to be dysregulated in HCV‐related HCC, clearly defined miRNA panels are still needed to distinguish HCV‐HCC from other liver diseases [15]. These panels could enhance diagnostic accuracy, prognostic assessment, and therapeutic targeting [16].

Among various miRNAs, miR‐1 has been notably associated with inflammatory conditions and the development and progression of multiple cancers due to its altered expression [17]. Evidence suggests that miR‐1 could act as a tumor activator in human HCC, where its inhibition leads to decreased cell proliferation, increased apoptosis, and reduced TEC migration and invasion [18]. Despite existing evidence, its role in HCV‐associated HCC is still ambiguous [19]. Some recent studies indicate that miR‐200b acts as a tumor suppressor and sensitizes lung cancer cells to CDDP, possibly via modulation of p70S6K1 expression [20]. miR‐200b has also been reported to exert tumor‐suppressive effects in HCC through the modulation of DNMT3a expression [21]. The inhibitory effect of miR‐200b‐3p on tumor angiogenesis was confirmed in another study, which also highlights its potential therapeutic value in suppressing HCC progression [22]. Despite evidence supporting its role in HCC, the exact contribution of miR‐200b to HCV‐induced HCC has not been clearly defined. In the case of miR‐320d, studies suggest that exosomal miR‐320d contributes to tumor metastasis and angiogenesis through GNAI1 downregulation and JAK2/STAT3 activation [23]. Further, evidence suggests that its upregulation leads to reduced proliferation and invasion of HCC cells [24]. Nonetheless, the function of miR‐320d in the context of HCV‐induced hepatocellular carcinoma is not yet well understood. In addition to these miRNA, miRNA‐346 has also been shown to promote proliferation, migration, and invasion in liver cancer cells [25]. Similarly, the involvement of miR‐451a in cancer progression has been documented in several malignancies, including osteosarcoma, colorectal carcinoma, and breast cancer [26]. However, their role in HCV‐related HCC remains to be evaluated.

Based on the above observations, this study aimed to compare the relative expression of selected miRNAs (miR‐1, miR‐200b, miR‐320d, miR‐346, and miR‐451a) in GII (HCV patients), GIII (HCV‐HCC patients), and GIV (HCC patients) with GI (healthy control) to find out the association of selected miRNAs with selected biomarkers and determine their prognostic ability. For this, RT‐PCR was used to validate their expression patterns in relation to NF‐κB and IL‐6 across different study groups. Compared to GI, a significant downregulation of miR‐1, miR‐346, and miR‐451 was observed in both GII and GIII. Conversely, miR‐200b, miR‐320d, NF‐κB, and IL‐6 were markedly upregulated in GIII. Further bioinformatics analysis revealed strong interactions between the selected miRNAs, biomarkers, and their target mRNAs.

## 2 Methods

### 2.1 Data Mining and Selection of miRNA

The role of miR‐1, miR‐200b, miR‐320d, miRNA‐346, and miR‐451a was explored by using data mining. All the studies exploring the role of miR‐1, miR‐200b, miR‐320d, miRNA‐346, and miR‐451a were studied, and all the diseases with a close association with selected miRNA were retrieved. Data mining techniques were also employed to elucidate the roles of miR‐1, miR‐200b, miR‐320d, miRNA‐346, and miR‐451a in HCV induced HCC patients.

### 2.2 Sample Collection

To determine the expression profile of the selected miRNA panel, serum samples were collected from various hospitals across Punjab, Pakistan. This study included 100 participants and was classified into four groups: (1) GI group: 20 healthy people; (2) GII group: 20 patients with HCV infection; (3) GIII group: 40 patients with HCV‐induced HCC; and (4) GIV group: 20 patients with HCC (Unknown etiology). Blood samples were obtained from cancer patients who were admitted to different Pakistani cancer hospitals. Consent was duly obtained from all patients. The ethical committee of UOL provided the approval for the current study. For the present study, CONSORT guidelines and guidelines by Assel et al. (2018) were followed for proper analysis, reporting, and interpretation of clinical research [27].

### 2.3 Inclusion and Exclusion Criteria

All individuals in the present study were older than 18 years. The healthy individuals did not have a history of liver disease, nor did they have any other serious illnesses affecting the kidneys, lungs, or other essential organs. GIII (HCV‐induced HCC patients) and GIV groups (HCC patients) were diagnosed with at least two imaging modalities (hepatic ultrasonography plus magnetic resonance imaging, computed tomography (CT), or both) and with advanced‐stage cancer. This study excluded patients who had a previous history of other cancers.

### 2.4 Quantitative Identification of HCV RNA

In this study, quantitative identification of HCV RNA was conducted by using the COBAS AmpliPrep/COBAS TaqMan HCV Quantitative Tests, v2.0, on the Cobas 6800 system (Roche Diagnostics). HCV‐positive patients were defined as those with a viral load greater than 4500 IU/mL. A high viral load was defined as > 800,000 IU/mL, whereas a low viral load was defined as < 800,000 IU/mL.

### 2.5 RNA Purification and cDNA Synthesis

Whole RNA from serum samples was isolated using the Quick‐cfRNA Serum & Plasma Kit (Catalog # R1059) (ZYMO RESEARCH). The isolated RNA was quantified with the help of Qubit^TM^ Catalog # Q32852. cDNA of the total RNA was generated by using the cDNA Synthesis Kit (Thermo Scientific #K1622).

### 2.6 RT‐PCR Amplification

Expression analysis of dysregulated miRNAs was performed by RT‐PCR. RT‐PCR amplification of miRNAs, including miR‐1, miR‐200b, miR‐3200d, miR‐346, and miR‐451a, and selected biomarkers by using Maxima SYBR Green/ROX qPCR Master Mix (2X) (Thermo Scientific #K0221). As a reference gene, Glyceraldehyde‐3‐phosphate dehydrogenase (GAPDH) was used. The sequences of primers are mentioned in the following table (Table 1).

**TABLE 1:** Sequences of primers.

miRNAs	Nucleotide sequence (5'−3')	bp length
miR‐1	F‐ TGGGAAACATACTTCTTTAT	20
	R‐ TGAGATACATACTTCTTTAC	20
miR‐200b	F‐CAGCCGTGGCCATCTTACT	20
	R‐CCGCCGTCATCATTACCAGG	19
miR‐320d	F‐AGTGCTTCCATGTTTGAGTGT	21
	R‐CACACTCAAACATGGAAGCAC	21
miR‐346	F‐GGTCTCTGTGTTGGGCGTC	19
	R‐CCCAGCCCCTGCCTCCTT	18
miR‐451a	F‐TTGGGAATGGCAAGGAAACC	20
	R‐ATGGTTCTCTTGCTATACCCAG	22
NF‐κB	F‐GCACCCTGACCTTGCCTATT	20
	R‐CTGCTTGGCGGATTAGCTCT	20
IL‐6	F‐CTGCGATGGAGTCAGAGGAA	20
	R‐TTCTCTTTCGTTCCCGGTGG	20
GAPDH	F‐CGACCACTTTGTCAAGCTCA	20
	R‐AGG GGT CTA CAT GGC AAC TG	20

### 2.7 Data Analysis

To calculate the expression profile of selected miRNA and biomarkers, the 2^−∆∆Ct^ method was used [28].

### 2.8 Statistical Evaluation

To perform the statistical analysis, GraphPad Prism 8.0.2 was used. A one‐way analysis of variance (ANOVA), Tukey's multiple comparison analyses, was performed to find out the statistical comparisons of the data. To assess the correlation between miRNA expression and immune‐related biomarkers, we used Spearman's correlation analysis. ROC analysis was performed to estimate the sensitivity (true positive rate) and specificity (false positive rate) of the defined groups. Further AUC was also determined. In this investigation, two‐tailed statistical analysis was performed, and *p*‐value ≤ 0.05 was considered. Statistical analyses were conducted in accordance with the SAMPL guidelines.

### 2.9 Bioinformatics Analysis

We used different bioinformatics tools, including themiRDB database [29] and miRWalk 3.0 [30], to find out candidate genes interacting with selected miRNAs.

### 2.10 miRNA‐mRNA Network Construction

We used the TargetScanHuman 7.2 (https://www.targetscan.org/vert_72/) [31] to screen out interacting mRNAs with our selected miRNAs. Cytoscape (version 3.10.1) was employed to analyze the interactions [32].

## 3 RESULTS

### 3.1 Role of Selected miRNA in Different Diseases

**FIGURE 1:**
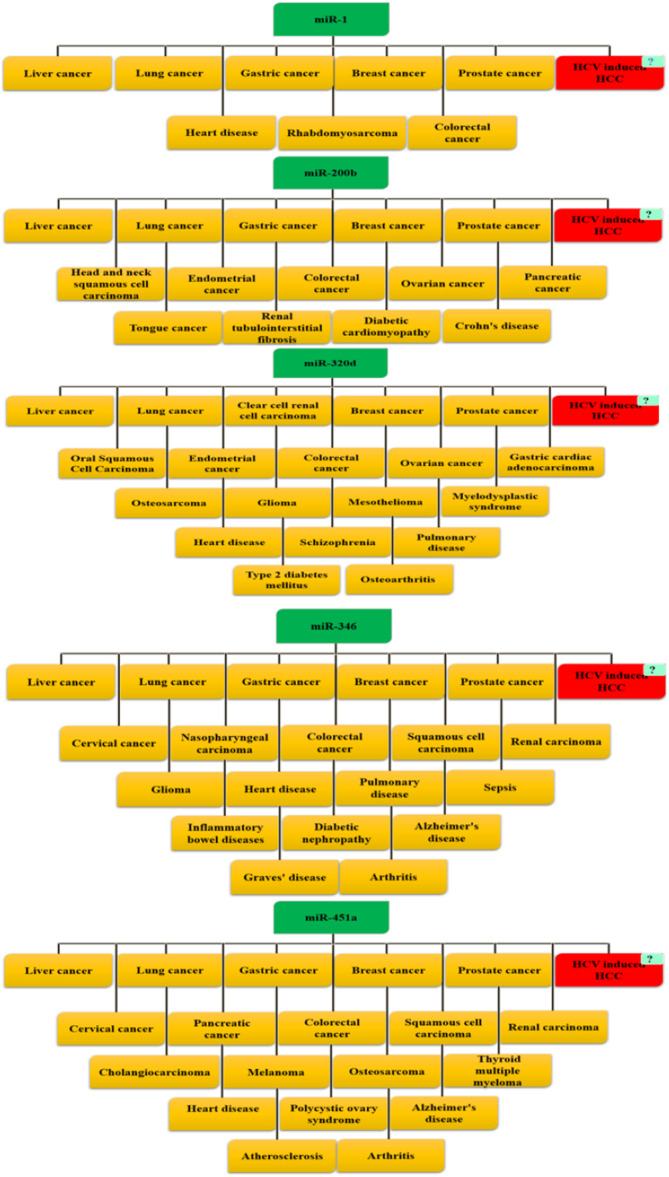
The schematic diagram illustrates the reported involvement of miR‐1, miR‐200b, miR‐320d, miR‐346, and miR‐451a across a wide range of human diseases. Each microRNA (shown in green boxes) is linked to multiple malignancies (yellow boxes), including liver, lung, gastric, breast, prostate, colorectal, and other site‐specific cancers, as well as non‐malignant conditions such as cardiovascular, metabolic, inflammatory, neurological, and autoimmune disorders. HCV‐induced hepatocellular carcinoma (HCC) is highlighted in red to emphasize its recurrent association across all five microRNAs.

An extensive literature review and systematic database mining were performed to assess the pathological significance of selected miRNA across a broad range of human diseases. Data mining was also performed to find out all selected miRNAs (miR‐1, miR‐200b, miR‐320d, miR‐346, and miR‐451a) reported against HCV‐HCC. miR‐1 is associated with inflammatory conditions and the development and progression of multiple cancers, including liver, lung, gastric, prostate, colorectal, breast cancers, rhabdomyosarcoma, as well as heart disease [33]. miR‐200b is involved in cancers of the liver, lung, stomach, breast, ovary, pancreas, prostate, colorectum, endometrium, head and neck squamous cell carcinoma, tongue cancer, and is also implicated in renal tubulointerstitial fibrosis, diabetic cardiomyopathy, and Crohn's disease. miR‐320d has been reported to be involved in liver, lung, prostate, and breast cancers; oral squamous cell carcinoma; endometrial cancer; clear cell renal cell carcinoma; colorectal and ovarian cancers; gastric cardiac adenocarcinoma; osteosarcoma; glioma; mesothelioma; and myelodysplastic syndrome, as well as non‐malignant conditions including heart disease, schizophrenia, pulmonary disease, type 2 diabetes mellitus, and osteoarthritis. miR‐346 is implicated in several malignancies, including liver, lung, breast, prostate, cervical, nasopharyngeal, colorectal, squamous cell, renal carcinomas, and glioma, while in non‐malignant diseases it is associated with heart disease, pulmonary disease, sepsis, inflammatory bowel diseases, diabetic nephropathy, Alzheimer's disease, Graves' disease, and arthritis. miR‐451a is known to be involved in breast, lung, liver, gastric, colorectal, prostate, pancreatic, esophageal, and ovarian cancers, as well as osteosarcoma, glioma, leukemia, cardiovascular disease, type 2 diabetes mellitus, pulmonary disease, sepsis, inflammatory diseases, and neurological disorders. Notably, miR‐1, miR‐200b, miR‐320d, miR‐346, and miR‐451a have not been reported in association with HCV‐induced HCC (HCV‐HCC), and their roles in HCV‐HCC remain to be investigated (Figure 1).

### 3.2 Serum Sample Collection

To find out the relative expression of the selected miRNAs, serum samples were collected from patients classified in four groups (GI‐GIV). The demographical characteristics of different study groups, GI‐GIV, are highlighted in Table 2.

**TABLE 2:** Demographic characteristics of the different study groups.

Variables	GI	GII	GIII	GIV	*p* value
**Age**	36.7 ± 14.72	47.3 ± 12.99	55.4 ± 9.28	47.3 ± 12.99	0.00
**< 50**	7 (35.00)	8 (40.00)	7 (17.50)	8 (40.00)	
**≥ 50**	13 (65.00)	12 (60.00)	33 (82.50)	12 (60.00)	
**Gender**					0.00
**Female**	12 (60.00)	9 (45.00)	11 (72.50)	5 (25.00)	
**Male**	8 (40.00)	11 (55.00)	29 (27.50)	15 (75.00)	

### 3.3 Relative Expression of Dysregulated miRNAs

**FIGURE 2:**
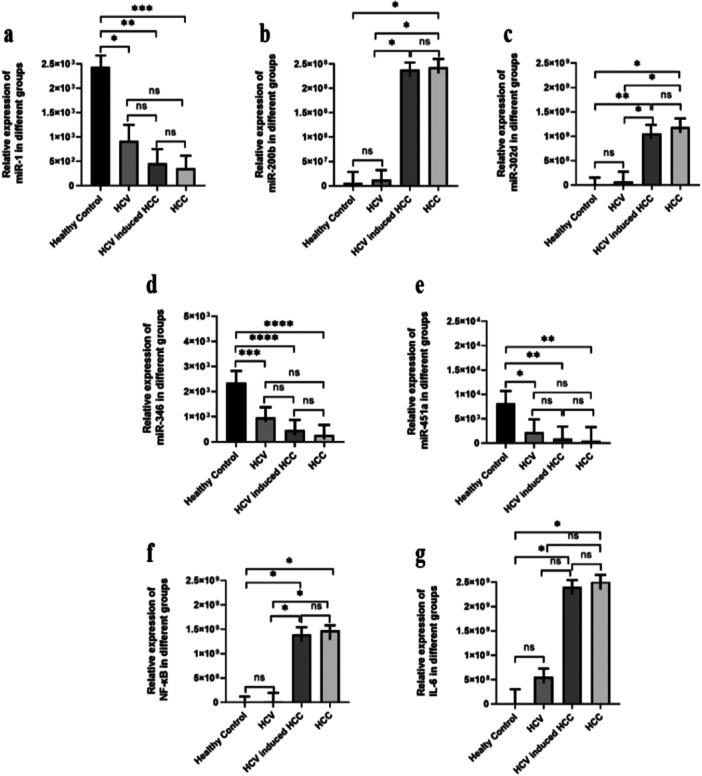
Relative expression of selected microRNAs and inflammatory markers across study groups. (a) miR‐1, (b) miR‐200b, (c) miR‐320a, (d) miR‐346, (e) miR‐451a, (f) NF‐κB, and (g) IL‐6 expression levels were analyzed in Healthy Control, HCV, HCV‐induced HCC, and HCC groups. Data are presented as relative expression levels. miR‐1, miR‐346, and miR‐451a showed significant downregulation in disease groups compared to healthy controls, whereas miR‐200b and miR‐320a were significantly upregulated in HCV‐induced HCC and HCC groups. Inflammatory markers NF‐κB and IL‐6 exhibited increased expression in HCV‐induced HCC and HCC, indicating enhanced inflammatory activity during disease progression. Statistical significance is indicated as *p < 0.05, **p < 0.01, ***p < 0.001, ****p < 0.0001, and ns = not significant.

In the present study, the expression level of selected miRNAs and immune biomarkers was evaluated among the groups (GI, GII, GIII, and GIV). The viral load in Group II (GII) ranged from > 4500 to < 800,000 IU/mL, whereas in Group III (GIII) it ranged from ≥ 800,000 to 10,000,000 IU/mL. In Group I (GI), no viral load was detected, that is, < 150 IU/mL. Then the expression levels of the GII, GIII, and GIV groups were compared with the GI. One‐way ANOVA and Tukey's multiple comparison test were used for statistical analysis. The present study showed that miR‐1, miR‐451a, and miR‐346 were notably downregulated in GII (*p* = 0.02, *p* = 0.0004, and *p* = 0.01), GIII (*p* = 0.001, *p* < 0.001, and *p *= 0.002), and GIV (*p* = 0.001, *p* < 0.001, and *p* = 0.001) in comparison to GI group (Figure 1). The notable upregulated expression in GIII was observed against NF‐κB, IL‐6, miR‐320d, and miR‐200b (*p* = 0.04, *p* = 0.02, *p* = 0.03, and *p* = 0.04) and GIV (*p* = 0.03, *p* = 0.00, *p* = 0.02, and *p* = 0.03) when compared with GI. High expression levels in GIII and GIV were also found against miR‐200b, miR‐320d, and NF‐κB (*p* = 0.04, *p* = 0.03, and *p* = 0.04) (*p* = 0.0145 and *p* = 0.02) when they were compared with the GII group. However, no notable significance was found against selected miRNAs among GIII and GIV when compared (Figure 2).

**FIGURE 3:**
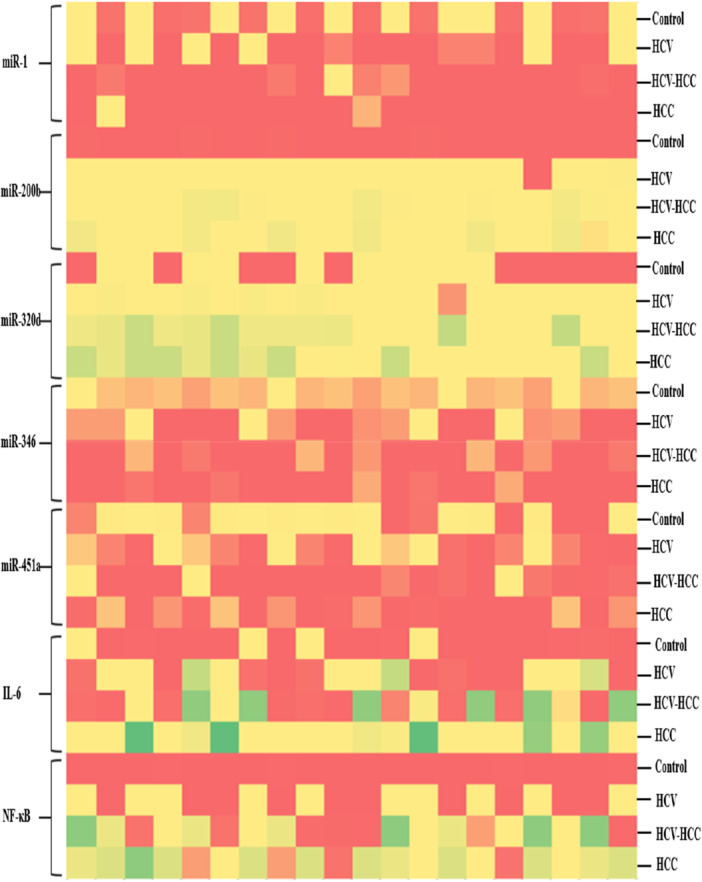
Heatmap plot: Differential expression of selected miRNAs and biomarkers within the defined groups. Green and red colors represent high and low expression.

The relative expression levels of the selected microRNAs (miRNAs) across the four study groups—Group I (GI), Group II (GII), Group III (GIII), and Group IV (GIV)—were visualized using a heatmap. To generate this heatmap, the ΔCT (delta cycle threshold) values for each miRNA and immune‐related biomarker were calculated. The ΔCT values, which represent the normalized expression levels of the target miRNAs relative to a reference gene, were used to indicate the relative abundance or suppression of each miRNA within the sample groups (Figure 3). The heatmap was constructed using Microsoft Excel, which allowed for a clear and intuitive visual representation of expression trends across the groups. In the heatmap, color gradients were applied to depict varying levels of miRNA regulation. Specifically, higher expression levels (indicating upregulation) were represented by darker shades of green, while lower expression levels (indicating downregulation) were illustrated using darker shades of red. Intermediate expression levels were shown with lighter shades of these colors, providing a continuous visual scale for comparison. This visual representation facilitated the identification of distinct expression patterns and group‐specific regulatory differences, thereby supporting the interpretation of the potential role of each miRNA and biomarker in disease progression or immune modulation.

### 3.4 miRNAs Correlation With Immune‐Related Biomarkers in Study Groups

Furthermore, Spearman's correlation analyses were used to quantitatively calculate the expression levels of IL‐6 and NF‐κB to assess their association with selected miRNAs. Negative correlation was found between IL‐6 and miR‐1 (*r* = −0.6896, *p* = 0.0043), but positive correlation was observed between miR‐451a and NF‐κB (*r* = 0.5941, *p* = 0.0171) in the GIII group. The results indicate that miR‐451a upregulation may stimulates the NF‐κB‐mediated inflammatory responses while miR‐1 may inhibit IL‐6 expression. However, no significant correlation between miRNAs was found in GII and GIII groups (Table 3).

**TABLE 3:** Spearman rank correlations of miRNAs and immune biomarkers in HCV, HCV‐HCC, and HCC patients (*r*/*p*).

Variables	miR‐1	miR‐200b	miR‐320d	miR‐346	miR‐451a
**GII**					
**IL‐6**	*r* = 0.1478	*r* = −0.02027	*r* = 0.1308	*r* = −0.1917	*r* = 0.1501
	*p* = 0.5798	*p* = 0.9364	*p* = 0.6147	*p* = 0.4460	*p* = 0.5751
**NF‐κB**	*r* = 0.05773	*r* = 0.006501	*r* = 0.2849	*r* = 0.09341	*r* = −0.1399
	*p* = 0.8335	*p* = 0.9796	*p* = 0.2652	*p* = 0.7037	*p* = 0.6017
**GIII**					
**IL‐6**	*r* = −0.6896	*r* = 0.3834	*r* = −0.1094	*r* = 0.3761	*r* = 0.1754
	*p* = 0.0043	*p* = 0.1163	*p* = 0.6726	*p* = 0.1239	*p* = 0.5109
**NF‐κB**	*r* = 0.08983	*r* = −0.02309	*r* = 0.04845	*r* = −0.2731	*r* = 0.5941
	*p* = 0.7385	*p* = 0.9276	*p* = 0.4263	*p* = 0.2580	*p* = 0.0171
**GIV**					
**IL‐6**	*r* = 0.3341	*r* = −0.3243	*r* = 0.1838	*r* = 0.2946	*r* = −0.02875
	*p* = 0.2045	*p* = 0.1892	*p* = 0.4757	*p* = 0.2353	*p* = 0.9158
**NF‐κB**	*r* = −0.1557	*r* = −0.2538	*r* = −0.07039	*r* = 0.06687	*r* = −0.2378
	*p* = 0.5603	*p* = 0.3094	*p* = 0.7856	*p* = 0.7856	*p* = 0.3706

### 3.5 ROC Analysis

#### 3.5.1 Diagnostic Potential of Selected miRNAs, NF‐κB, and IL‐6 in GII in Comparison to GI

**FIGURE 4:**
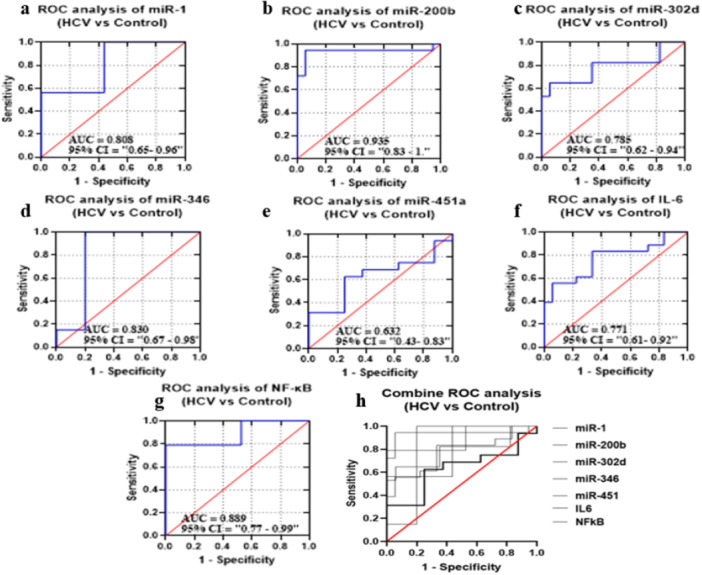
ROC curves were constructed to evaluate the diagnostic performance of (a) miR‐1, (b) miR‐200b, (c) miR‐302d, (d) miR‐346, (e) miR‐451a, (f) IL‐6, and (g) NF‐κB. The area under the curve (AUC) with corresponding 95% confidence intervals (CI) is indicated in each panel. miR‐200b exhibited the highest diagnostic accuracy (AUC = 0.935), followed by NF‐κB (AUC = 0.889), miR‐346 (AUC = 0.830), miR‐1 (AUC = 0.808), miR‐302d (AUC = 0.785), and IL‐6 (AUC = 0.771), whereas miR‐451a showed comparatively lower performance (AUC = 0.632). (h) Combined ROC analysis demonstrates the collective diagnostic potential of all studied markers, indicating improved discrimination between HCV patients and healthy controls. The red diagonal line represents the line of no discrimination (AUC = 0.5).

ROC curves of candidate miRNAs, IL‐6, and NF‐κB were drawn to distinguish GII group from G1 group, as shown in Figure 4. The AUC values were 0.808, 0.935, 0.785, 0.83, 0.632, 0.771, and 0.889, corresponding to the selected miRNA and biomarkers. The *p* values were also statistically significant (*p* = 0.0029, *p* < 0.0001, *p* = 0.0045, *p* = 0.004, *p* = 0.0054, and *p* < 0.0001); however, miR‐451a showed no significance value (*p* = 0.2). All miRNAs and biomarkers represent sensitivity and specificity within the specific range. A combination of miRNA panel and biomarkers, however, improved specificity and sensitivity and AUC by 70.16%, 53.323%, and 0.6762, respectively (*p*‐value < 0.0001) (Figure 4) (Supplementary Table 1).

#### 3.5.2 Diagnostic Potential of the Candidate miRNAs and Selected Biomarkers in GIII in Comparison to GI Group

**FIGURE 5:**
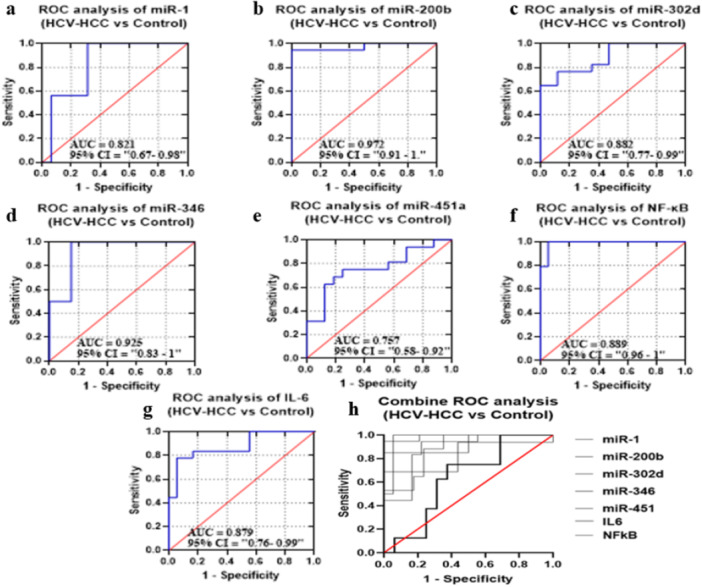
ROC curves and AUC for miRNAs and biomarkers to compare GIII (HCV‐HCC patients) and GI (healthy controls) groups. (a–g) ROC curves showing diagnostic performance of individual markers: (a) miR‐1, (b) miR‐200b, (c) miR‐302d, (d) miR‐346, (e) miR‐451a, (f) NF‐κB, and (g) IL‐6. For each panel, the area under the curve (AUC) and 95% confidence interval (CI) are indicated. The red diagonal line represents the line of no discrimination. (h) Combined ROC curve analysis of all studied biomarkers, demonstrating their collective diagnostic performance in distinguishing HCV‐HCC patients from controls. Sensitivity is plotted against 1 − specificity for all curves.

ROC analysis was performed for selected miRNA and biomarkers to compare GIII from GI (Figure 5). The calculated values of AUC were 0.828 = miR‐1, 0.972 = miR‐200b, 0.882 = miR‐320d, 0.92 = miR‐346, 0.757 = miR‐451a, 0.879 = IL‐6, and 0.988 = NF‐κB. The *p*‐value was statistically relevant for all (*p* < 0.0001). All miRNAs and biomarkers showed sensitivity (100% to 44.44%) and specificity (99.44% to 75%) for the GIII group (HCV‐HCC patients). A combined miRNA panel and biomarkers improved overall sensitivity, specificity, and AUC by 84.68%, 58.06%, and 0.7118, respectively (*p*‐value < 0.0001) (Figure 5) (Supplementary Table 1).

#### 3.5.3 Diagnostic Potential of the Candidate miRNAs and Selected Biomarkers in GIII Group Compared to GII Group

**FIGURE 6:**
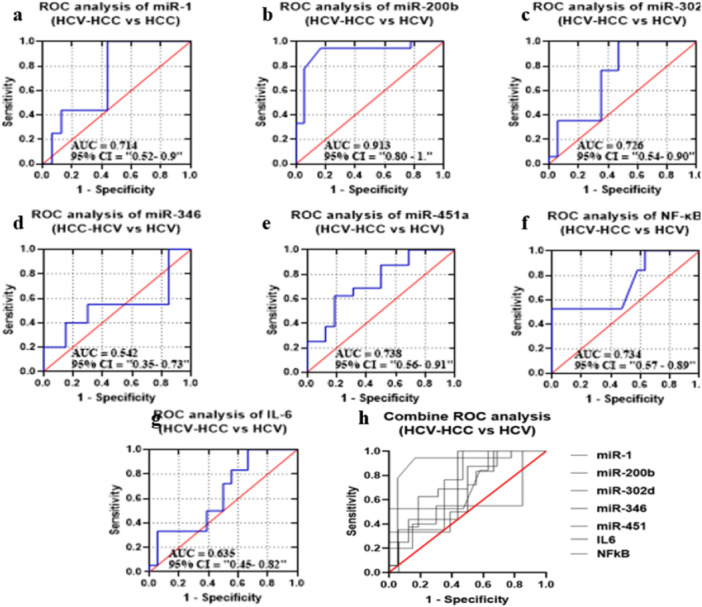
ROC curves and AUC for miRNAs and biomarkers to compare GIII (HCV‐HCC patients) and GII (HCV patients) groups. (a–g) ROC curves showing the diagnostic performance of individual biomarkers: (a) miR‐1, (b) miR‐200b, (c) miR‐302d, (d) miR‐346, (e) miR‐451a, (f) NF‐κB, and (g) IL‐6 in distinguishing HCV‐HCC patients from HCV patients without HCC. The area under the curve (AUC) and 95% confidence intervals (CI) are indicated in each panel. The red diagonal line represents the line of no discrimination.(h) Combined ROC curve analysis of all studied biomarkers, illustrating their overall diagnostic performance in differentiating HCV‐HCC from HCV patients. Sensitivity is plotted against 1 − specificity for all curves.

ROC analysis was performed to compare GIII to GII. AUC values were 0.7148, 0.9136, 0.7266, 0.5425, 0.7383, 0.6358, and 0.7341, corresponding to selected miRNA and biomarkers (Figure 6). The *p*‐value was statistically significant for all, that is, < 0.005. However, miR‐346 and IL‐6 were found to be statistically insignificant (*p* = 0.0241; *p* = 0.1639). The specificity (87.5% to 42.11%) and sensitivity (94.4% to 43.75%) for the GIII were evaluated. A combined miRNA panel and biomarkers improved sensitivity, specificity, and AUC by 53.233%, 59.68%, and 0.5748%, respectively (*p*‐value = 0.0418) (Figure 6) (Supplementary Table 1).

#### 3.5.4 Diagnostic Potential of the Candidate miRNAs and Selected Biomarkers in GIV group Compared to GI Group

**FIGURE 7:**
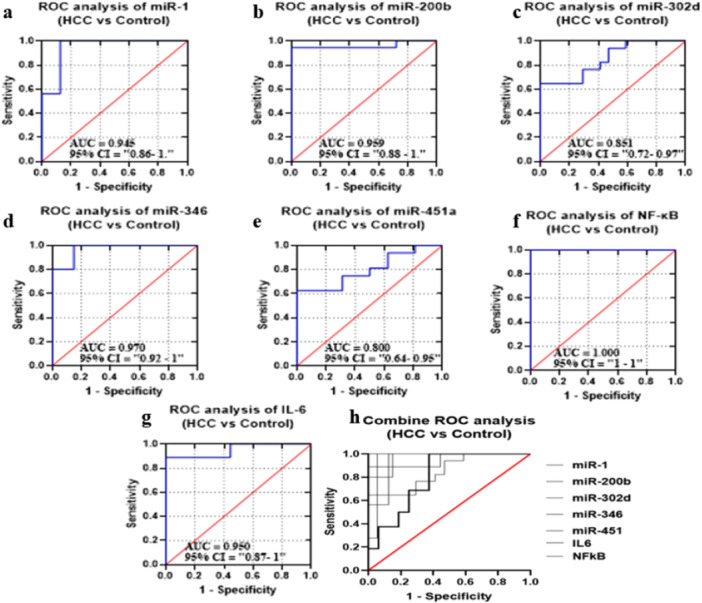
ROC curves and AUC for miRNAs and biomarkers to compare GIV (HCC patients) and GI (healthy controls) groups. (a–g) ROC curves illustrating the diagnostic performance of individual biomarkers: (a) miR‐1, (b) miR‐200b, (c) miR‐302d, (d) miR‐346, (e) miR‐451a, (f) NF‐κB, and (g) IL‐6 in distinguishing HCC patients from controls. The area under the curve (AUC) and corresponding 95% confidence intervals (CI) are shown in each panel. The red diagonal line represents the line of no discrimination.(h) Combined ROC curve analysis of all investigated biomarkers, demonstrating their overall diagnostic accuracy in differentiating HCC patients from healthy controls. Sensitivity is plotted against 1 − specificity for all curves.

ROC curves of candidate miRNAs and biomarkers were drawn to compare GIV from GI (Figure 7). AUC values of selected miRNA and biomarkers were 0.9453, 0.9599, 0.8512, 0.97, 0.8008, 0.9506, 1.000, and 0.6934, respectively. The *p*‐values were also found to be statistically significant (*p* < 0.0005). High sensitivity was observed against all miRNAs and biomarkers (94% to 37%) in GIV group (HCC patients). A collective miRNA and biomarkers showed improved sensitivity, specificity, and AUC 87.10%, 55.65%, and 0.6934, respectively (*p*‐value < 0.0001) (Figure 7) (Supplementary Table 1).

#### 3.5.5 Diagnostic Potential of the Candidate miRNAs and Selected Biomarkers in GIV Group Compared to GII Group

**FIGURE 8:**
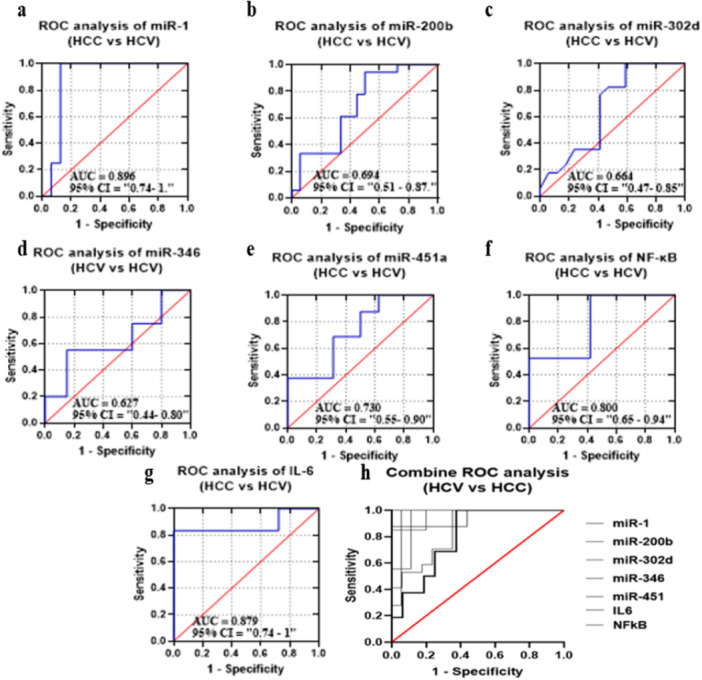
ROC curve analyses evaluating the diagnostic performance of selected microRNAs and inflammatory markers in distinguishing GIV (HCC patients) and GII (HCV patients) groups. (a) miR‐1, (b) miR‐200b, (c) miR‐302d, (d) miR‐346, (e) miR‐451a, (f) NF‐κB, and (g) IL‐6. The area under the curve (AUC) and 95% confidence intervals (CI) are indicated in each panel. The red diagonal line represents the line of no discrimination. (h) Combined ROC curve analysis of all tested miRNAs biomarkers showing their comparative diagnostic performance. Sensitivity is plotted against 1 − specificity.

ROC curves of candidate miRNAs and biomarkers were drawn to discriminate G4 group from G2 group, as displayed in Figure 8. The AUC values were 0.8906, 0.6944, 0.6644, 0.6275, 0.7305, 0.8796, and 0.8006, respectively. The *p*‐values were also found to be statistically significant for miR‐1, miR‐200b, miR‐451a, IL‐6, and NF‐κB (*p* = 0.0002, *p* = 0.0462, *p* = 0.0262, *p* < 0.0001, and *p* = 0.0015). However, miR‐346 and miR‐346 were not significant (*p* = 0.1018 and *p* = 0.1677). High sensitivity and specificity were observed against all miRNAs and biomarkers (ranging from 100% to 43.75%) and (ranging from 87.5% to 27.78%) for GIV group (HCC patients). A collective miRNA and biomarkers showed improved sensitivity, specificity, and AUC by 60.48%, 686.5%, and 0.5639, respectively (*p*‐value = 0.0821) (Figure 8) (Supplementary Table 1).

#### 3.5.6 Diagnostic Potential of the Candidate miRNAs and Selected Biomarkers in GIV Compared to GIII

**FIGURE 9:**
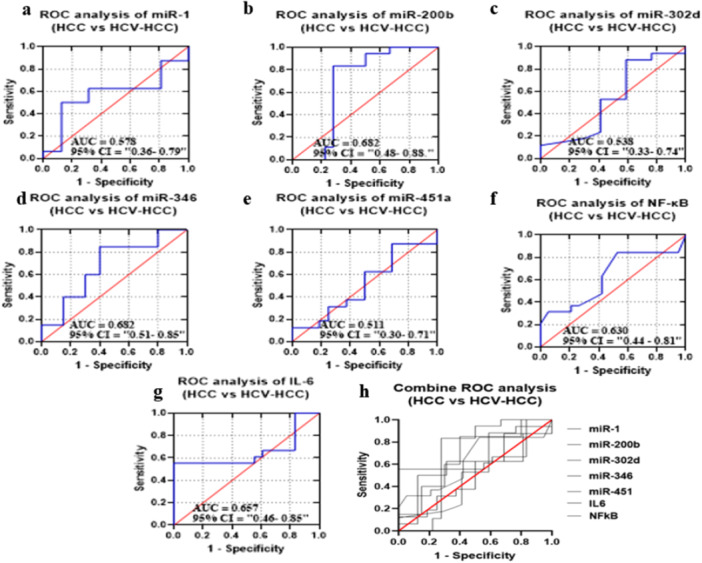
ROC curve analyses evaluating the diagnostic performance of selected microRNAs and inflammatory markers in distinguishing GIV (HCC patients) and GIII (HCV‐HCC patients) groups. (a) miR‐1, (b) miR‐200b, (c) miR‐320d, (d) miR‐346, (e) miR‐451a, (f) NF‐κB, and (g) IL‐6. The area under the curve (AUC) and 95% confidence intervals (CI) are indicated in each panel. The red diagonal line represents the line of no discrimination. (h) Combined ROC curve analysis of all tested miRNA and biomarkers showing their comparative diagnostic performance. Sensitivity is plotted against 1 − specificity.

ROC curves of candidate miRNAs and biomarkers were drawn to discriminate GIV from GIII (Figure 9). AUC values against selected candidates were 0.5781, 0.6821, 0.5381, 0.6825, 0.5117, 0.6574, and 0.6302. The *p*‐value was found to be statistically relevant for miR‐346 (*p* = 0.0483). All miRNAs and biomarkers represent sensitivity and specificity in a specific range (Sensitivity = 85% to 61.11%; Specificity = 72.222% to 50%) for G4 (HCC patients). The sensitivity of 81.45%, specificity of 27.42%, and an AUC of 0.5090, with a *p*‐value of 0.8070, were demonstrated against the selected candidates (Figure 9) (Supplementary Table 1).

### 3.6 miRNA–mRNA Network Construction

**FIGURE 10:**
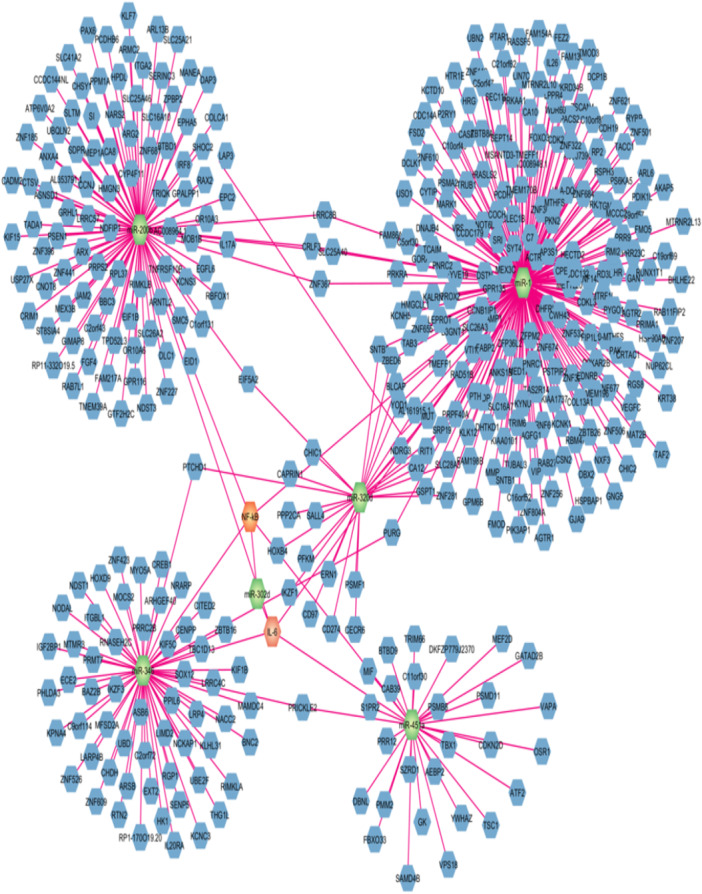
miRNA–mRNA regulatory network construction using Cytoscape. The green octagon represented miRNAs, blue octagon represented target mRNAs, and orange octagon represented immune‐related biomarkers in network.

To predict the possible mRNA targets of miRNAs (miR‐1, miR‐200b, miR‐320d, miR‐346, and miR‐451a), we used the miRDB database (https://www.mirbase.org/) [29] and TargetScanHuman7.2 (https://www.targetscan.org/vert_72/) [31]. It was revealed that miR‐1, miR‐200b, miR‐320d, miR‐346, and miR‐451a may interact with a large number of genes, including IL‐6 and NF‐κB. For the construction of the miRNA‐mRNA network, Cytoscape (version 3.10.1) was used [32]. The target genes interacting with miR‐1, miR‐200b, miR‐3200d, miR‐346, and miR‐451a are shown in Figure 10.

## 4 Discussion

HCC is the most prevalent type of liver cancer [34], while HCV infection is one of the causative agents of HCC development [1]. Chronic inflammation is a primary mechanism connecting HCV infection to the development of HCC. Persistent HCV infection triggers continuous immune system activation, causing liver injury, fibrosis, and cancer development [35].

IL‐6 [36] and NF‐κB are key inflammatory mediators in the progression of HCV‐associated liver disease. IL‐6 promotes cell proliferation, survival, and angiogenesis in liver cells. Elevated IL‐6 levels are found in patients with CHC and are even higher in those with HCC, where they correlate with poor prognosis and increased tumor burden [37, 38]. NF‐κB regulates genes involved in inflammation and cell survival, and remains persistently active during chronic hepatic injury. NF‐κB continuous activation contributes to fibrosis, progressive liver damage, and HCC by promoting a microenvironment conducive to cancer development [39, 40]. Clinical studies have demonstrated that both IL‐6 and NF‐κB levels are elevated in CHC patients as compared to healthy individuals. HCV‐induced HCC patients often show further alterations in these biomarkers due to tumor‐induced inflammation. However, similar biomarker changes are observed in HCC resulting from other etiologies, such as chronic alcohol consumption or metabolic disorders. Therefore, IL‐6 and NF‐κB are not specific biomarkers for HCV‐related HCC. However, the IL‐6 and NF‐κB specificity as individual biomarkers for HCV‐induced HCC is limited, since similar inflammatory activation occurs in HCC arising from non‐viral causes, including alcohol‐induced and metabolic liver diseases HCC [35, 39]. In another study, it is reported that HCV infection triggers an NF‐κB–dependent upregulation of miR‐221 [41]. HCV‐induced NF‐κB activation not only supports viral replication but also modulates the host immune response, promoting liver inflammation. Similarly, patients with chronic hepatitis caused by hepatitis C virus exhibit increased interleukin‐6 concentrations [42]. A separate study demonstrated that HCV infection is associated with increased NIK expression and reduced levels of both HNF4A and miR‐122, representing NF‐κB and IL‐6 as better diagnostic markers against both HCV and HCV induced HCC [43].

miRNA aberrant expression has also been reported in various kinds of malignancies, including HCC induced by HCV. These miRNAs are ~22‐nt long, non‐coding, endogenous RNAs that play a main role in gene regulation [8]. Various miRNAs are considered as a key target against various cancers [10]. Among them, miR‐1 generally acts as a tumor suppressor [44]. Its downregulation is associated with increased proliferation, migration, and invasion in several cancers, including liver, lung, and bladder cancers [45]. miR‐200b suppresses epithelial‐to‐mesenchymal transition, invasion, and metastasis [46]. It often acts as a tumor suppressor in cancers such as breast, lung, and liver [47]. miR‐320d functions variably, but in many cancers like HCC, its overexpression can inhibit proliferation and invasion, indicating a tumor‐suppressive role [24]. miR‐346 often functions as an oncogene. It is overexpressed in various cancers, promoting proliferation, migration, and invasion, including in liver and prostate cancers [48]. While miR‐451a typically acts as a tumor suppressor [49]. It is involved in inhibiting cancer cell growth and metastasis in cancers [50] like colorectal, breast, and osteosarcoma [51]. All these miRNAs play a significant role in different cancers, including HCC, but their exact role in HCV‐induced HCC needs to be elucidated. In this study, literature mining and bioinformatic analysis were performed to screen out miRNAs, as well as immune‐regulatory biomarkers IL‐6 and NF‐κB, to investigate their role in HCV and HCV‐HCC. Furthermore, RT‐PCR was used to evaluate their expression profile in the defined groups (GI‐GIV): GI (healthy controls), GII (HCV patients), GIII (HCV‐induced HCC patients), and GIV (with unknown cause of cancer) study groups. The expression level of miR‐1, miR‐346, and miR‐451 was observed to be downregulated in GII, GIII, and GIV groups (*p* = 0.0005, *p* < 0.0001, and *p* = 0.0006, respectively), when compared with control (GI). However, the expression of the remaining miRNA was found to be upregultaed in GIII and GIV groups as compared with GI (miR‐200b: *p* = 0.0039, miR‐320d: *p* = 0.0010, NF‐κB: *p* = 0.0025, and IL‐6: *p* = 0.0111). However, no significant relevance was observed within selected miRNAs when GII was compared with GI, and when GIII was compared with GIV (Figure 2).

Furthermore, a significant correlation between five candidate miRNAs and two immune‐related biomarkers was found. We identified that two miRNAs were correlated with biomarkers. In GIII, miR‐451a (*r* = 0.5941, *p* < 0.0171) was found to have a positive association with NF‐κB, while miR‐1 (*r* = −0.6896, *p* < 0.0043) has a negative association with IL‐6. Consistent with our finding, Li et al. (2019) reported that miR‐451a promotes NF‐κB‐mediated inflammatory responses [52], whereas Chen et al. (2022) described that miR‐1 can inhibit IL‐6 expression and suppresses oxidative stress and inflammatory responses [53]. The results indicate that miR‐451a upregulation may stimulates the NF‐κB‐mediated inflammatory responses while miR‐1 may inhibit IL‐6 expression. So, we assume that these candidate miRNAs may directly or indirectly show a close association; however, further experimentation is required to test this possibility. Moreover, we identified the diagnostic potential of miR‐1 (AUC = 0.8281), miR‐200b (AUC = 0.9722), miR‐320d (AUC = 0.8824), and miR‐346 (AUC = 0.925) for G3 group and miR‐1 (AUC = 0.9453), miR‐200b (AUC = 0.9599), miR‐320 (AUC = 0.8512), miR‐346 (AUC = 0.97), and miR‐451a (AUC = 0.8008) for G4 group, as shown in Supplementary Table 1. This was particularly true in the case of miR‐1, miR‐200b, and miR‐346, which showed relevant AUC values, showing their high diagnostic potential as biomarkers. Furthermore, bioinformatic analysis was performed to find out the interaction between selected miRNAs and mRNAs. Our analysis indicated that a wide array of mRNAs are capable of interacting with selected miRNAs, as shown in Figure 9. We compiled a comprehensive list of these miRNAs and predicted their target genes using the TargetScan tool. These interactions were visualized by constructing a circRNA–miRNA–mRNA interaction network using Cytoscape software (version 3.10.1). We found that our candidate miRNAs have good diagnostic potential for HCV‐HCC.

The present study has certain limitations, such as a comparatively small sample size; a population‐based study could confirm the data; and further research experimentation is required to investigate whether the above miRNA panel can effectively distinguish HCV‐HCC from other cancers. However, the findings of this cross‐sectional study highlight the potential clinical utility of a serum‐based miRNA panel as a non‐invasive diagnostic tool for Hepatitis C virus (HCV)‐induced HCC. Future studies should focus on validating this miRNA signature in larger, multicenter, and ethnically diverse cohorts to establish its diagnostic robustness and generalizability across different populations and HCV genotypes. The miRNA panel (miR‐1, miR‐200b, miR‐320d, miR‐346, and miR‐451a) reported in the present study can serve as diagnostic biomarkers for early detection of HCV‐HCC and as therapeutic targets for HCV‐HCC treatment.

## 5 Conclusions

The selected panel of miRNA have serves as diagnostic biomarkers against HCV‐HCC.

## Author Contributions

**Aqsa Ikram, Badr Alzahrani, Mohammed Bourhia,** and **Yasmeen Ishaq:** conceptualization, formal analysis, investigation, supervision, first draft writing. **Tawaf Ali Shah, Gamal A. Shazly, Yasmeen Ishaq, Qaiser Farid Khan,** and **Turki M. Dawoud:** conceptualization, methodology, formal analysis, manuscript writing.

## Ethics Statement

The present research was approved by the Ethical Committee of the Institute of Molecular Biology and Biotechnology (IMBB), University of Lahore, Lahore, Pakistan. For all research procedures and publication of this study, informed consent was taken from participants.

## Conflicts of Interest

The authors declare no conflicts of interest.

## Transparency Statement

The lead authors, Badr Alzahrani,and Aqsa Ikram, affirm that this manuscript is an honest, accurate, and transparent account of the study being reported; that no important aspects of the study have been omitted; and that any discrepancies from the study as planned (and, if relevant, registered) have been explained.

## Supporting information

Supporting File

## Data Availability

The data that support the findings of this study are available from the corresponding author upon reasonable request.
