# Supplementary material for: Serum‐Based miRNA Panel as Diagnostic Biomarkers for Hepatitis C Virus‐Induced Hepatocellular Carcinoma: A Cross‐Sectional Study
Source: Health Sci Rep. 2026 Apr 15;9(4):e72377. doi: 10.1002/hsr2.72377 (PMC13084151; doi:10.1002/hsr2.72377)
Supplement: Supplementary file 1 — Supporting File [file HSR2-9-e72377-s001.docx]

Supplementary Material

# Supplementary Data

**Supplementary Table 1: ROC analysis**

| **miRNA** | **Sensitivity** | **Specificity** | **95% CI** | **AUC** | **Cut-off** | **Std. Error** | **P value** |
| --- | --- | --- | --- | --- | --- | --- | --- |
| **HCV vs Control** | | | | | | | |
| miR-1 | 81.25 | 56.25 | 0.6552 to 0.9620 | 0.8086 | 3.7 | 0.07826 | 0.0029 |
| miR-200b | 94.44 | 66.67 | 0.8324 to 1.000 | 0.9352 | 6.1 | 0.05245 | <0.0001 |
| miR-320d | 82.35 | 64.71 | 0.6225 to 0.9484 | 0.7855 | 4.7 | 0.08315 | 0.0045 |
| miR-346 | 80 | 80 | 0.6771 to 0.9829 | 0.83 | 6.0 | 0.07801 | 0.0004 |
| miR-451a | 68.75 | 62.5 | 0.4310 to 0.8346 | 0.6328 | 3.1 | 0.103 | 0.2 |
| IL-6 | 83.3 | 66.67 | 0.6143 to 0.9289 | 0.7716 | 5.0 | 0.08026 | 0.0054 |
| NF-κB | 100 | 47.37 | 0.7797 to 0.9987 | 0.8892 | 4.7 | 0.05585 | <0.0001 |
| Combined panel | 70.16 | 53.323 | 0.6099 to 0.7425 | 0.6762 |  | 0.03381 | <0.0001 |
| **HCV-HCC vs Control** | | | | | | | |
| miR-1 | 81.25 | 68.75 | 0.6739 to 0.9824 | 0.8281 | 5.0 | 0.07869 | 0.0015 |
| miR-200b | 94.44 | 100 | 0.9164 to 1.000 | 0.9722 | 9.4 | 0.0285 | <0.0001 |
| miR-320d | 82.35 | 58.82 | 0.7711 to 0.9936 | 0.8824 | 4.1 | 0.05676 | 0.0001 |
| miR-346 | 80 | 85 | 0.8383 to 1.000 | 0.925 | 6.5 | 0.04421 | <0.0001 |
| miR-451a | 75 | 68.75 | 0.5859 to 0.9297 | 0.7578 | 4.3 | 0.0877 | 0.0129 |
| IL-6 | 88.89 | 44.44 | 0.7654 to 0.9938 | 0.8796 | 3.3 | 0.05826 | <0.0001 |
| NF-κB | 78.95 | 94.74 | 0.9639 to 1.000 | 0.9889 | 7.3 | 0.01279 | <0.0001 |
| Combined panel | 84.68 | 58.06 | 0.6470 to 0.7767 | 0.7118 |  | 0.03309 | <0.0001 |
| **HCC vs Control** | | | | | | | |
| miR-1 | 81.25 | 87.5 | 0.8656 to 1.000 | 0.9453 | 6.8 | 0.04067 | <0.0001 |
| miR-200b | 94.44 | 83.3 | 0.8818 to 1.000 | 0.9599 | 8.8 | 0.03984 | <0.0001 |
| miR-320d | 76.47 | 64.71 | 0.7244 to 0.9780 | 0.8512 | 4.1 | 0.06469 | 0.0005 |
| miR-346 | 100 | 85 | 0.9268 to 1.000 | 0.97 | 8.0 | 0.02205 | <0.0001 |
| miR-451a | 75 | 37.5 | 0.6435 to 0.9581 | 0.8008 | 1.2 | 0.08027 | 0.0037 |
| IL-6 | 88.89 | 55.56 | 0.8792 to 1.000 | 0.9506 | 4.4 | 0.03645 | <0.0001 |
| NF-κB | 100 | 94.74 | 1.000 to 1.000 | 1 | 9.4 | 0.00 | <0.0001 |
| Combined panel | 87.10 | 0.5565 | 0.6265 to 0.7603 | 69.34 |  | 0.03415 | <0.0001 |
| **HCV-HCC vs HCV** | | | | | | | |
| miR-1 | 43.75 | 87.5 | 0.5256 to 0.9041 | 0.7148 | 3.1 | 0.09654 | 0.0382 |
| miR-200b | 94.4 | 66.67 | 0.8074 to 1.000 | 0.9136 | 2.7 | 0.05415 | <0.0001 |
| miR-320d | 82.35 | 52.94 | 0.5485 to 0.9047 | 0.7266 | 3.5 | 0.09087 | 0.0241 |
| miR-346 | 55 | 70 | 0.3520 to 0.7330 | 0.5425 | 2.5 | 0.09721 | 0.6456 |
| miR-451a | 68.75 | 32.15 | 0.5658 to 0.9108 | 0.7383 | 6.9 | 0.08802 | 0.0215 |
| IL-6 | 72.2 | 44.44 | 0.4501 to 0.8216 | 0.6358 | 1.6 | 0.09477 | 0.1639 |
| NF-κB | 84.21 | 42.11 | 0.5708 to 0.8973 | 0.7341 | 2.6 | 0.08329 | 0.0136 |
| Combined panel | 53.23 | 59.68 | 0.5021 to 0.6475 | 0.5748 |  | 0.03710 | 0.0418 |
| **HCC vs HCV** | | | | | | | |
| miR-1 | 43.75 | 87.5 | 0.7460 to 1.000 | 0.8906 | 3.1 | 0.07379 | 0.0002 |
| miR-200b | 61.1 | 66.67 | 0.5181 to 0.8708 | 0.6944 | 2.7 | 0.08997 | 0.0462 |
| miR-320d | 76.47 | 58.82 | 0.4744 to 0.8544 | 0.6644 | 3.5 | 0.09694 | 0.1018 |
| miR-346 | 55 | 85 | 0.4492 to 0.8058 | 0.6275 | 3.5 | 0.09099 | 0.1677 |
| miR-451a | 87.5 | 50 | 0.5553 to 0.9057 | 0.7305 | 3.7 | 0.08939 | 0.0262 |
| IL-6 | 100 | 27.78 | 0.7493 to 1.000 | 0.8796 | 2.7 | 0.06649 | <0.0001 |
| NF-κB | 84.21 | 57.89 | 0.6569 to 0.9442 | 0.8006 | 4.2 | 0.0733 | 0.0015 |
| Combined panel | 60.48 | 68.65 | 0.4896 to 0.6382 | 0.5639 |  | 0.03790 | 0.0821 |
| **HCC vs HCV-HCC** | | | | | | | |
| miR-1 | 62.5 | 68.75 | 0.3663 to 0.7900 | 0.5781 | 3.1 | 0.1081 | 0.451 |
| miR-200b | 83.3 | 72.22 | 0.4828 to 0.8814 | 0.6821 | 5.5 | 0.1017 | 0.0619 |
| miR-320d | 82.35 | 41.18 | 0.3351 to 0.7410 | 0.5381 | 2.3 | 0.1036 | 0.7048 |
| miR-346 | 85 | 60 | 0.5134 to 0.8516 | 0.6825 | 4.5 | 0.0863 | 0.0483 |
| miR-451a | 62.5 | 50 | 0.3059 to 0.7176 | 0.5117 | 50 | 0.105 | 0.91 |
| IL-6 | 61.11 | 38.89 | 0.4613 to 0.8535 | 0.6574 | 6.1 | 0.1 | 0.1066 |
| NF-κB | 63.16 | 57.89 | 0.4472 to 0.8132 | 0.6302 | 2.1 | 0.09335 | 0.17 |
| Combined panel | 81.45 | 27.42 | 0.4367 to 0.5813 | 0.5090 |  | 0.03690 | 0.8070 |
